# Emergency Department Boarding and Mortality: A Systematic Review and Proportional Meta‐Analysis

**DOI:** 10.1002/hsr2.73400

**Published:** 2026-11-04

**Authors:** Gerardo Altamura, Mario Cesare Nurchis, Silvia Martinelli, Annarita Panebianco, Gabriele Giubbini, Marcello Covino, Francesco Franceschi, Gianfranco Damiani

**Affiliations:** ^1^ Medical Management, IDI‐IRCCS ‐ FLMM Rome Italy; ^2^ Department of Health Sciences and Public Health Section of Hygiene, Università Cattolica del Sacro Cuore, Largo Francesco Vito 1 Rome Italy; ^3^ Department of Woman and Child Health and Public Health Fondazione Policlinico Universitario Agostino Gemelli IRCCS Rome Italy; ^4^ Health Management, Fondazione Policlinico Universitario Agostino Gemelli IRCCS Rome Italy; ^5^ Emergency Medicine Department Fondazione Policlinico Universitario Agostino Gemelli‐IRCCS, Università Cattolica del Sacro Cuore di Roma, Largo A. Gemelli 8 Rome Italy

**Keywords:** crowding, ED boarding, emergency department, in‐hospital mortality, meta‐analysis, patient outcomes

## Abstract

**Background and Aims:**

Emergency department (ED) crowding, worsened by ED boarding (EDB), is defined as the stay of admitted patients in the emergency department due to the unavailability of inpatient beds. This occurrence poses a significant public health challenge, leading to adverse patient outcomes. Despite efforts to improve patient care pathways, the relationship between EDB and mortality is still contentious. This study aims to synthesize and compare reported in‐hospital mortality (IHM) among ED patients across studies stratified by ED boarding duration thresholds, with particular attention to shorter boarding durations.

**Methods:**

A systematic review and proportional meta‐analysis were conducted following PRISMA guidelines. MEDLINE, EMBASE, Web of Science, and Scopus were searched up to April 2024. Pooled mortality proportions were estimated using random‐effects models with DerSimonian–Laird estimation and Freeman–Tukey double arcsine transformation. Heterogeneity was assessed using I^2^. Leave‐one‐out analyses were performed to assess the robustness of subgroup estimates, with particular attention to longer boarding thresholds.

**Results:**

Twenty studies published between 2007 and 2024 were included. The overall pooled in‐hospital mortality proportion was 8.3% (95% CI: 7.6%–9.0%; I^2^ = 99.9%). Subgroup analyses showed pooled mortality estimates of 10.2% for early admission/no boarding, 4.4% for 1‐h boarding, 5.9% for 2‐h boarding, 4.8% for 4‐h boarding, 13.5% for 6‐h boarding, 20.5% for 8‐h boarding, 21.8% for 12‐h boarding, and 11.8% for 24‐h boarding. Shorter boarding thresholds were not associated with higher pooled mortality estimates compared with early admission/no boarding. Although longer boarding thresholds showed higher pooled mortality estimates, the 8‐, 12‐, and 24‐h subgroup estimates were based on few studies and were materially influenced by Verma et al. 2021. After excluding this study, the pooled mortality estimates decreased to 7.9%, 3.9%, and 4.0% for the 8‐, 12‐, and 24‐h subgroups, respectively. Given the extreme heterogeneity, these pooled estimates require cautious interpretation. Because different studies contributed to different threshold‐specific pools, the estimates are descriptive and should not be interpreted as evidence of a threshold or dose‐response relationship.

**Conclusion:**

This study highlights the possible association between prompt admissions, targeted interventions, and outcomes in the context of ED boarding. Improving ED operations and reducing boarding times within these durations remain important organizational objectives. Further research with standardized methodologies is essential to fully understand and address this complex issue. However, the present pooled proportions do not establish a boarding‐duration threshold, dose‐response relationship, or causal association, and their interpretation is limited by extreme heterogeneity (I^2^ = 99.9%).

## Highlights

- Prolonged ED boarding times (8–24 h) showed higher pooled in‐hospital mortality estimates.
- Shorter boarding thresholds of 1–6 h were not associated with higher pooled mortality estimates.
- Longer boarding thresholds showed higher pooled mortality estimates, but evidence was based on few studies.
- Meta‐analysis suggests an association between boarding duration and mortality rates.
- Reducing ED boarding durations remains an important organizational objective.

## 1 Introduction

### 1.1 Background

Emergency departments (ED) are finding it increasingly difficult to deliver high‐quality care due to crowding [1, 2]. Crowding is a risk factor for poor quality of care and adverse events after evaluation in the ED [3, 4] and the Institute of Medicine identified ED crowding as a major public health challenge [5]. This appears to be an international problem [6, 7, 8] that has raised concern in health care and is expected to become increasingly problematic with population growth and an aging population whose life expectancy is increasing [9]. In recent decades, EDs have attempted to reduce crowding by improving patient flow and processes, such as triage, diagnosis, and treatment, that affect the flow of care [10, 11]. However, these measures had little effect on reducing crowding because they did not address the source of the problem at the system level [12]. The literature indicates that ED boarding time is a major cause of ED crowding [2, 9, 10, 13, 14, 15]. ED boarding (EDB) is defined as the stay of patients in the ED after admission has been accepted to the hospital, due to the absence of inpatient beds [12, 16]. Both boarding and subsequent crowding have been definitively associated with adverse patient outcomes and threats to patient safety [12, 17, 18, 19, 20]. EDB has been associated with several negative patient‐oriented outcomes, from worse satisfaction to higher inpatient mortality rates [17, 18, 19, 20]. In Italy EDB has become a particularly pressing issue, especially in the wake of the COVID‐19 pandemic [21]. The surge in patient volumes due to delayed treatments and a growing demand for emergency care driven by an aging population and a higher prevalence of chronic diseases has led to longer boarding times. Compounding these challenges are workforce shortages and burnout among healthcare professionals, which hinder hospitals' ability to manage patient flow effectively [22]. Additionally, there is a renewed focus from policymakers on improving emergency care services, sparking discussions around potential reforms [23]. Increased public awareness of healthcare challenges has further amplified demands for better patient care in EDs [24]. Together, these factors create a critical moment for addressing the issue of boarding in Italy's healthcare system.

Several recent articles have identified safety issues related to EDB, including adverse events, higher rates of pneumonia following ventilator use [25], delays in antibiotic administration [26], delays in pain medication administration [4, 27, 28], lower patient satisfaction [29], prolonged times to disposition among patients with acute asthma [30], and higher complication rates for cardiovascular events [28] higher mortality rates for patients in general and particularly those in intensive care units (ICU) [25, 31]. There are also higher rates of medication errors and preventable adverse events [32, 33]. Therefore, EDB appears to be a central problem of public health, especially risk management, because it is associated with multiple adverse events and potentially dangerous patient outcomes.

Although scientific studies on ED crowding have increased, only a few studies have shown that ED crowding increases length of stay, hospital mortality, and ICU admission [31, 34, 35, 36]. EDB can have substantial consequences that lead to delayed interventions. For example, early onset therapy in severe sepsis leads to a significant improvement in in‐hospital mortality [37]. In addition, EDB has become a significant barrier to skilled critical care [16] and can compromise patient safety when there are not enough staff in the ED, increasing medical errors [38]. However, there are conflicting data on the rarely investigated association between delayed hospitalization and outcome [39, 40].

While the literature in this area of research has been growing in recent years, only a few studies have demonstrated a link between EDB and in‐hospital mortality [41, 42, 43].

A systematic review of the literature [35], while not finding robust evidence of an increase in EDB‐related in‐hospital mortality (IHM), nevertheless identified a low association between EDB and IHM, which would need further studies to be confirmed. In addition, no recent systematic review of the literature has analysed the relationship between EDB and IHM, focusing on the waiting time duration impact on adverse outcomes. Therefore, the purpose of this study was to synthesize and analyze reported in‐hospital mortality among emergency department patients in relation to ED boarding duration, by updating and consolidating the available evidence to date.

## 2 Materials and Methods

### 2.1 Study Design and Search Strategy

A systematic review of the literature was performed by querying the relevant scientific electronic databases, including MEDLINE, EMBASE, ISI Web of Science, and Scopus from the commencement to April 2024 to retrieve peer‐reviewed articles. No time filter has been applied. Only primary studies were included in the analysis.

The population, exposure, comparison, outcome (PECO) framework [44] was used to outline the following research question: “How does reported in‐hospital mortality vary according to ED boarding duration?”. The population consists of patients accessing the ED. The considered exposure is based on the concept of boarding, which could be defined as the practice of holding patients in the ED while awaiting hospital admission due to lack of available inpatient beds. The comparator is the absence of boarding while the main outcome is mortality.

The Preferred Reporting Items for Systematic Reviews and Meta‐Analyses (PRISMA) checklist [45] was adopted to build up a comprehensive search strategy (Supporting Information Table S1). This work was recorded on PROSPERO, International prospective register of systematic reviews (ID: CRD42024578999). For each scientific database, controlled descriptors and specific keywords were identified. Then, a search string was created interpolating Medical Subject Headings (MeSH) and free‐text words, such as “Boarding,” “Waiting time,” “emergency unit,” “emergency department,” “outcome,” “mortality,” “survival” with Boolean operators. Citation tracking was conducted to find missing articles, thus enriching the search strategy.

The full electronic search strategies for all databases, including filters and search terms, are provided in the Supporting Information to ensure reproducibility.

### 2.2 Study Selection

Study selection was managed using Microsoft Excel to facilitate duplicate removal and screening. No automation tools were used in the screening process.

Three authors (G.A., M.C.N., and M.C.) independently screened articles. The inclusion criteria were defined as follows: primary studies, focused on patients accessing ED, comparing mortality rates with and without boarding. The inclusion was limited to articles written in English and original research articles (i.e., no abstracts, review articles, editorials, or letters were included). The search strategy was further restricted by age (i.e., patients older than 18 years old), type of disease (i.e., no time‐sensitive diseases), and available primary studies databases (i.e., studies not reporting numerosity). Time‐sensitive diseases were identified by a group of emergency medicine health specialists based on their clinical knowledge, generalizable as medical conditions that require rapid diagnosis and treatment to prevent serious complications, worsening of the condition, or even death.

Two authors (G.A. and S.M.) independently evaluated the eligibility criteria, with a third author (M.C.N.) consulted in case of any discrepancies. Following the removal of duplicate articles, and following the predetermined inclusion and exclusion criteria, three researchers (G.A., S.M., and M.C.N.) independently conducted the first screening by assessing the titles and abstracts. Subsequently, these same individuals reviewed the full text of each study to assess potential eligibility. Any discrepancies during both screening phases were resolved through discussion with a fourth author (G.G.) about the article's inclusion and exclusion criteria.

### 2.3 Quality Assessment

The Risk of Bias in Non‐randomized Studies of Interventions (ROBINS‐I) tool was used to rate the risk of bias for non‐randomized included studies. This tool assesses seven domains: risk of bias from confounding, selection of participants, classification of interventions, deviations from intended interventions, missing data, measurement of outcomes, and selection of the reported results. A proposed judgment about the risk of bias arising from each domain is generated by an algorithm, based on answers to the signaling questions. Judgment can be “Low,” “Moderate,” or “High” risk of bias, or can express “Some concerns” [46]. The ROBINS‐I tool provides categorical risk of bias assessments. However, it does not mandate study exclusion based on these assessments; rather, it assists in evaluating the overall quality of evidence [47]. The overall certainty of evidence was considered in light of study design limitations, risk of bias, and heterogeneity across studies, although a formal GRADE assessment was not performed. This absence of a formal certainty‐of‐evidence assessment is acknowledged as a limitation.

### 2.4 Data Extraction

Data extraction was completed by three investigators independently (G.A., S.M., and M.C.N.). An electronic data extraction spreadsheet was set up to extract the following variables: first author's name, year of publication, study design, country of origin, number of patients included, type of hospital, patients' disease, mortality without boarding, mortality with boarding, patients' characteristics, time to death. The primary outcome was in‐hospital mortality. All mortality data consistent with this outcome domain were extracted as reported in each study, including different measures, time points, and analytic formats. When multiple estimates were available, the most directly attributable to the defined exposure groups were selected. Extracted variables included study characteristics (author, year, country, design, hospital type, population/disease, study period), exposure definition (boarding time), and sample size. Outcome data included mortality with and without boarding, with corresponding numerators and denominators, and mean age when available. Funding sources were extracted when reported. No assumptions or imputations were made for missing or unclear data; only reported information was used.

Mortality outcomes were treated as dichotomous variables and synthesized using pooled prevalence/proportion estimates with corresponding 95% confidence intervals (CIs).

### 2.5 Data Analysis and Data Synthesis

Quantitative data synthesis was conducted by always keeping the in‐hospital mortality, in terms of the proportion of patients who die among those who present to the ED within a certain period, as the outcome of reference. Several cut‐offs were considered in the analysis: 1, 2, 4, 6, 8, 12, and 24 h.

Results from individual studies were systematically tabulated, including study characteristics and outcome data. Quantitative syntheses were presented using forest plots to visually display pooled estimates and corresponding 95% confidence intervals. Additional subgroup and sensitivity analyses were also illustrated graphically to facilitate interpretation of heterogeneity across studies.

Studies were allocated to each synthesis based on predefined boarding time cut‐offs. This process was guided by tabulating study‐level boarding duration data and matching each study to the most appropriate exposure category. When multiple time points were reported, data were assigned to all relevant predefined groups when applicable. Inclusion in each synthesis required that mortality outcomes could be directly attributed to the corresponding boarding category.

The unit of analysis was the study‐level mortality proportion within each reported boarding‐time threshold. When a study reported mortality data for more than one predefined boarding threshold, each threshold‐specific estimate was included in the corresponding subgroup. Therefore, some studies could contribute to more than one subgroup. These subgroup analyses should be interpreted as threshold‐specific descriptive syntheses rather than as statistically independent comparisons across mutually exclusive study populations. Accordingly, between‐subgroup comparisons were interpreted cautiously, and causal conclusions based on subgroup differences were avoided.

Pooled in‐hospital mortality proportions were estimated using random‐effects proportional meta‐analysis. Because several studies reported very low or near‐zero mortality proportions, the Freeman–Tukey double arcsine transformation was applied to stabilize variances before pooling. Estimates were pooled using inverse‐variance weighting, and between‐study variance was estimated using the DerSimonian–Laird τ^2^ estimator [48]. A proportional meta‐analysis synthesizes study‐specific outcome proportions rather than comparative effect estimates. Accordingly, the threshold‐specific pooled proportions were interpreted as descriptive summaries and not as estimates of a causal effect of boarding duration.

Seven included studies reported adjusted comparative estimates relating boarding duration to mortality or closely related hospitalization outcomes. These estimates were not pooled because exposure definitions, reference categories, patient populations, outcomes, and adjustment strategies were not sufficiently comparable for a meaningful comparative meta‐analysis.

Results were back‐transformed and expressed as pooled proportions with corresponding 95% confidence intervals and 95% prediction intervals. Confidence intervals for individual study proportions were calculated using the Clopper–Pearson method.

A 95% confidence interval estimates the range within which the true population is likely to fall, indicating our certainty based on sample data. In contrast, a 95% prediction interval predicts the range for future individual observations, accounting for both the uncertainty of the estimate and the variability among individual data points, typically resulting in a wider interval than the confidence interval.

In cases of significant heterogeneity, prediction intervals will be wider than confidence intervals, offering a more cautious approach to integrating uncertainty into the analysis [49]. Heterogeneity was inspected using the I^2^ statistic, with a threshold level for significant heterogeneity of 50% [50].

Methods for assessing publication bias, such as Egger's and Begg's tests alongside funnel plots, were originally designed for comparative data, assuming a bias toward publishing positive results over negative ones. While it is possible to apply these tests to proportional meta‐analyses, there is insufficient evidence to suggest they effectively account for such data. Additionally, the assumption of positive results being preferentially published may not hold true for proportional studies, given the absence of a standardized definition or consensus regarding positive outcomes in meta‐analyses of proportions [49].

Exploratory sensitivity analyses were performed to assess the influence of sparse or very small study‐level estimates. These analyses excluded studies with 10 or fewer mortality events and/or an observed mortality proportion below 0.015 to evaluate the robustness of the pooled estimate to sparse‐event studies and very low mortality proportions.

Statistical analyses were conducted adopting the R statistical software (version 4.5.2) [51]. Particularly, for the meta‐analysis of proportions, the “meta” package (version 5.0.0) was employed. Statistical significance was determined at a threshold of two‐sided *p*‐values < 0.05.

## 3 Results

### 3.1 Study Selection

The database search resulted in 2939 publications. After duplicate elimination, 1916 titles and abstracts were screened. A total of 71 articles were identified for a full‐text screening. After the full‐text examination, 51 papers were excluded since they did not fulfill the eligibility criteria. Thus, 20 articles were included in the systematic review and were considered for meta‐analysis (Figure 1).

**Figure 1:**
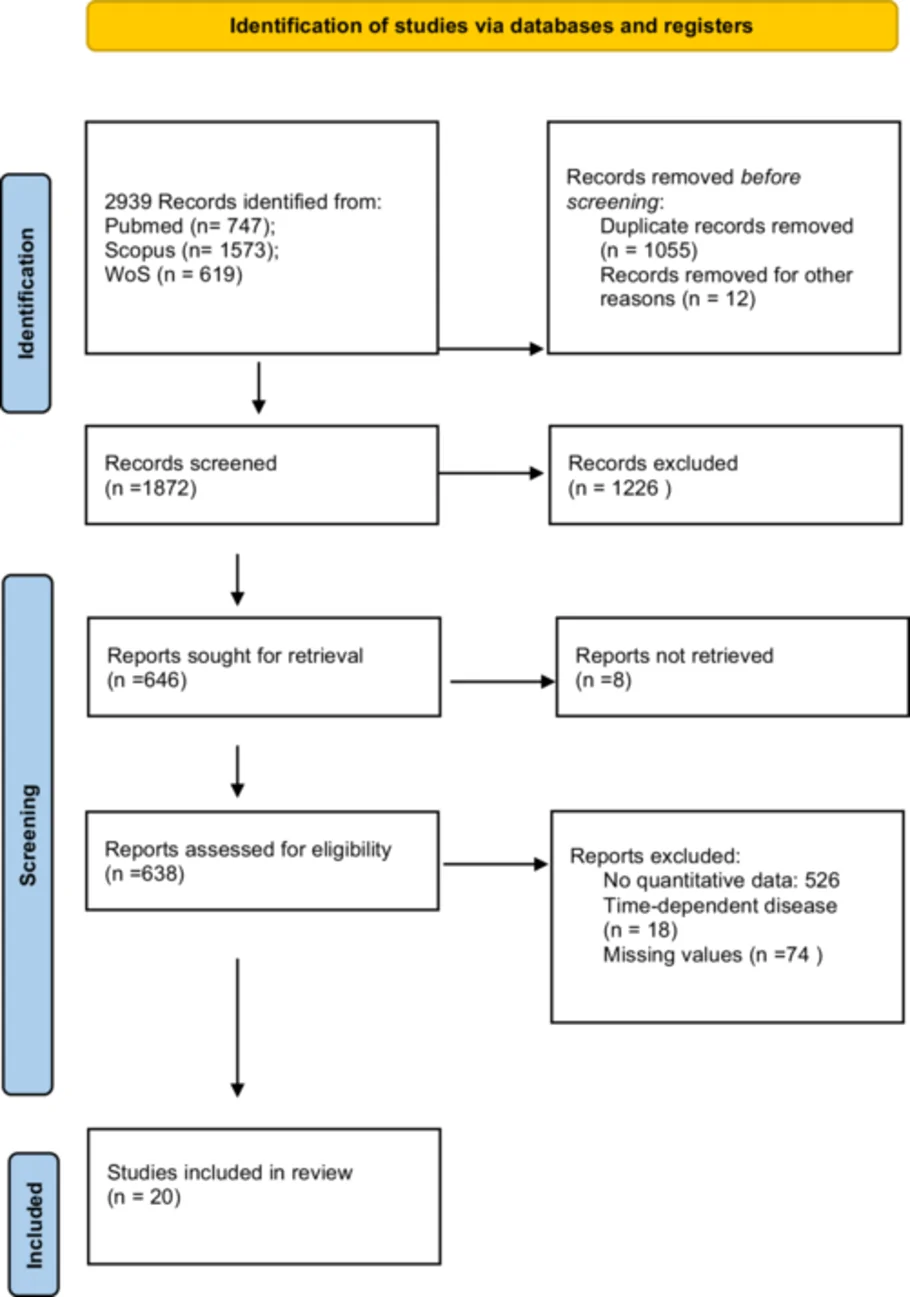
PRISMA 2020 flow diagram of study identification, screening, eligibility, and inclusion in the systematic review.

### 3.2 Study Characteristics

The selected manuscripts were published between 2009 and 2024, most of which from the USA [8], followed by China [2] and Taiwan [2], Canada, France, Greece, India, the Netherland, Switzerland, Thailand, and UAE (one each). Fifteen studies applied a retrospective cohort design, one case‐control, and four observational cross‐sectional. All the included articles reported numerosity and mortality of patients both with and without boarding. Eight studies evaluated all registered ED cases, seven studies referred only to critically ill patients, two studies only to sepsis, two studies reported data about necrotizing soft tissue infection, and one study only referred to appendicitis occurrences. Table 1 provides a summary of the main characteristics of each of the 20 publications.

**Table 1:** Characteristics of the included studies in the systematic review, including author, year, country, study design, disease, waiting time, sample size, and mean age.

Author	Year	Country	Design	Disease	Waiting time (hours)	Numerosity	Mean age
Bing‐Hua Y.U. [52]	2014	China	Retrospective cohort	Critically Ill	6	2279	57.3
Boulain T [53]	2020	France	Retrospective cohort	Critically Ill	4	68,876	63.8
Canal C [54]	2020	Switzerland	Retrospective cohort	Appendicitis	24	9224	36
Chalfin D.B. [25]	2007	USA	Cross‐sectional	All Emergency Department Accesses	6	50,322	58.4
Devkaran S [55]	2009	UAE	Case‐control	All Emergency Department Accesses	1	10,485	nr
Gardner K [56]	2022	USA	Retrospective cohort	Critically Ill	6, 12	1862	62.3
Groenland C.N. [57]	2019	Netherlands	Retrospective cohort	All Emergency Department Accesses	1, 4, 24	14,788	59
Guttmann A [58]	2011	Canada	Retrospective cohort	All Emergency Department Accesses	1, 4, 6, 8	13,934,542	nr
Hong Y.C. [43]	2009	Taiwan	Retrospective cohort	Necrotizing Soft Tissue Infection	8	195	55.3
Huang K.W. [59]	2024	Taiwan	Retrospective cohort	Critically Ill	6	1318	77
Intas G [60]	2012	Greece	Retrospective cohort	Critically Ill	6	200	57.8
Kongkaewpaisan N [61]	2020	USA	Retrospective cohort	Necrotizing Soft Tissue Infection	4	91	53
Lin S [62]	2021	China	Retrospective cohort	All Emergency Department Accesses	6	13,634	66.14
Lord K [63]	2018	USA	Cross‐sectional	All Emergency Department Accesses	4	31,426	59
Nesbitt J [64]	2021	USA	Retrospective cohort	Critically Ill	6	1959	60
Peltan I.D.[65]	2019	USA	Retrospective cohort	Sepsis	1	10,811	61.5
Puls H.A. [66]	2022	USA	Retrospective cohort	Critically Ill	6	5859	62
Singer A.J. [41]	2011	USA	Retrospective cohort	All Emergency Department Accesses	2, 6, 12, 24	41,256	53.1
Tantarattanapong S [67]	2021	Thailand	Cross‐sectional	Sepsis	1	600	78
Verma A [68]	2021	India	Cross‐sectional	All Emergency Department Accesses	4, 8, 12, 24	3429	62.3

### 3.3 Quality Assessment

The methodological quality evaluation of the included non‐randomized studies according to ROBINS‐I revealed varied levels of bias across different domains. Most studies proved a low risk of bias, due to confounding and deviations from intended interventions, suggesting robustness in these areas. However, a moderate risk of bias was notably present in several key domains, including participant selection, classification of interventions, missing data, and outcome measurement. Some studies [43, 52, 56, 57, 64, 66, 67, 68] particularly exhibited these moderate risks, indicating methodological concerns that could impact the reliability of their findings.

While many articles proved strong methodological rigor in addressing confounding variables, selecting participants, and implementing intended interventions with minimal deviations, some showed moderate risks of bias in areas such as the classification of interventions, handling of missing data, and measurement of outcomes. Despite these variations, most articles maintained a low risk of bias in transparently reporting their results. Overall, the articles represent a diverse range of approaches to studying the subject matter, highlighting both strengths and areas for improvement in non‐randomized study designs (see Table 2).

**Table 2:** Methodological quality evaluation of the included non‐randomized studies according to ROBINS‐I.

Author	Bias due to confounding domains relevant to the setting of the study	Bias in selection of participants into the study	Bias in classification of interventions	Bias due to deviations from intended interventions	Bias due to missing data	Bias in measurement of outcomes	Bias in selection of the reported results
Bing‐Hua	Low	Moderate	Moderate	Low	Moderate	Moderate	Low
Boulain	Low	Moderate	Low	Low	Moderate	Moderate	Low
Canal	Low	Low	Low	Low	Moderate	Low	Low
Chalfin	Low	Moderate	Low	Low	Moderate	Low	Low
Devkaran	Low	Moderate	Low	Low	Low	Moderate	Moderate
Gardner	Low	Moderate	Moderate	Low	Moderate	Moderate	Low
Groenland	Low	Moderate	Moderate	Low	Moderate	Low	Moderate
Guttmann	Low	Low	Low	Low	Moderate	Low	Low
Hong	Low	Low	Moderate	Low	Moderate	Moderate	Moderate
Huang	Low	Moderate	Low	Low	Moderate	Low	Low
Intas	Low	Moderate	Moderate	Low	Moderate	Low	Low
Kongkaewpaisan	Low	Moderate	Low	Low	Moderate	Low	Low
Lin	Moderate	Low	Low	Low	Moderate	Moderate	Low
Lord	Low	Low	Moderate	Low	Moderate	Low	Low
Nesbitt	Low	Moderate	Moderate	Low	Moderate	Moderate	Moderate
Peltan	Low	Low	Low	Low	Moderate	Low	Low
Plus	Low	Moderate	Low	Low	Moderate	Moderate	Moderate
Singer	Low	Low	Low	Low	Low	Low	Low
Tantarattanapong	Low	Moderate	Moderate	Low	Moderate	Low	Moderate
Verma	Low	Moderate	Low	Low	Moderate	Moderate	Moderate

### 3.4 Descriptive Synthesis of In‐Hospital Mortality by ED Boarding‐Duration Threshold

The overall pooled prevalence of mortality was 8.3% (95% CI: 7.6%–9%, *p* < 0.0001, *I* ^*2*^ = 99.9%) (Figure 2). The estimated 95% prediction intervals ranged from 4.5% to 13.4%.

**Figure 2:**
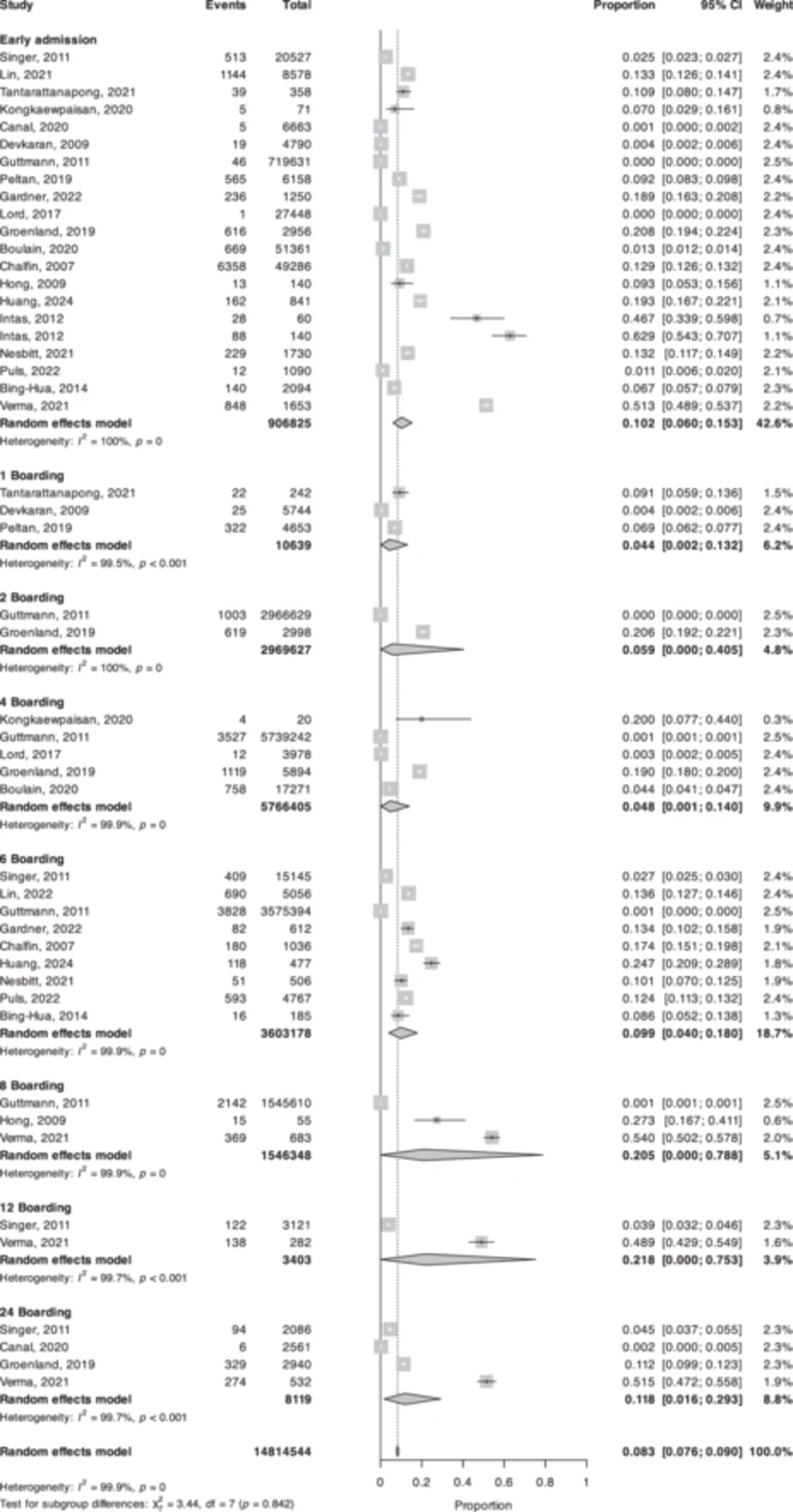
Forest plot illustrating mortality proportions across different boarding time thresholds compared with early admission.

Subgroup analysis showed lower pooled mortality estimates for shorter boarding thresholds than for longer thresholds; however, estimates for prolonged boarding durations, particularly 8, 12, and 24 h, were based on few studies and should therefore be interpreted cautiously (*p* for subgroup differences < 0.001).

Furthermore, subgroup analysis of patients waiting in the ED for 1, 2, 4, and 6 h before being moved to an inpatient bed showed no significant difference (*p* > 0.05) in mortality rates compared to patients who were promptly admitted without waiting (i.e., early admission/no boarding), implying that shorter boarding times within the above‐reported cut‐offs did not adversely affect patients' mortality.

For patients in the former group (i.e., early admission/no boarding), the pooled prevalence rate of mortality was 10.2% (95% CI: 0.6%–15.3%). For those in the latter group (i.e., boarding), the pooled prevalence rates of mortality were 4.4% (95% CI: 0.2%–13.2%), 5.9% (95% CI: 0.1%–40.5%), 4.8% (95% CI: 0.1%–14%), 13.5% (95% CI: 6.7%–22.3%), 20.5% (95% CI: 0.1%–78.8%), 21.8% (95% CI: 0.1%–75.3%), and 11.8% (95% CI: 1.6%–29.3%) for the 1‐, 2‐, 4‐, 6‐, 8, 12‐, and 24‐h cut‐off, respectively (Figure 2). To assess the robustness of subgroup estimates for longer EDB thresholds, a leave‐one‐out analysis was performed for the main meta‐analysis, with particular attention to the 8‐, 12‐, and 24‐h subgroups (Figure S2‐ Supporting Information). The original pooled mortality estimates for these subgroups were 20.5%, 21.8%, and 11.8%, respectively. These estimates were materially influenced by Verma et al. 2021. After excluding this study, the pooled mortality proportion decreased to 7.9% in the 8‐h subgroup, 3.9% in the 12‐h subgroup, and 4.0% in the 24‐h subgroup (Supporting Information Figure S3). The 12‐h estimate was reduced to a single remaining study, whereas the 8‐ and 24‐h estimates were based on only two and three studies, respectively. Confidence intervals remained wide, and heterogeneity remained substantial for the 8‐ and 24‐h subgroups. Because different studies contributed to the threshold‐specific pools, these subgroup estimates should be interpreted as descriptive summaries rather than direct comparisons between boarding durations.

Sensitivity analysis excluding 6 studies with a number of events (i.e., number of deaths) of 10 or less and/or an effect size lower than 0.015 (i.e., Kongkaewpaisan, Canal, Lord, Devkaran, Guttmann and Boulain) highlighted a higher overall pooled mortality rate of 16.8% (95% CI: 13.5%–20.4%, I2 = 99.6%, p for subgroup differences < 0.001). The estimated 95% prediction intervals ranged from 2% to 41.8%. In this sensitivity analysis, the pooled mortality proportions for the 8‐, 12‐, and 24‐h categories were numerically higher than the early admission/no‐boarding pool (Supporting Information Figure S1). Differently from the overall analysis, the 6‐h pool showed a lower numerical mortality proportion (12.2% 95% CI: 6.6%–19%) than the early admission/no‐boarding pool (16.8% 95% CI:13.5%–20.4%). Nonetheless, tests for subgroup analysis did not show statistically significant differences (*p* > 0.05) in mortality rates among the patients in these two groups. These are between‐pool descriptive differences and should not be interpreted as adjusted comparative effects.

## 4 Discussion

Unlike longer waiting times, shorter boarding durations (i.e., 1, 2, 4, and 6 h) were not associated with significantly higher reported in‐hospital mortality rates compared to patients who were admitted promptly from the ED (i.e., early admission/no boarding). Nevertheless, the present findings describe an elevated mortality estimate for patients enduring extended waiting periods [53]. This observation aligns with several studies that found no significant association between EDB and increased in‐hospital mortality or adverse outcomes, supporting the notion that EDB durations shorter than 6 h may not be significantly associated with patient mortality within the limits of study‐level analyses [69, 70, 71]. However, other literature indicates that prolonged EDB is significantly associated with higher IHM, particularly when delays exceed 6 h [39, 72]. These studies suggest that the reported association with EDB may become more apparent with such extended delays, highlighting the importance of prompt admissions. For instance, delays over 6 h have been correlated with increased mortality in ICU patients, emphasizing the critical nature of timely ICU admission [73]. Literature presents divergent findings regarding the association between ED crowding and quality of care in both ICU and non‐ICU patient populations [56, 74, 75]. Selected studies employed varied definitions for boarding, with durations ranging from 1 to 24 h. Notably, a majority, making up roughly 70% of all studies, defined a boarding duration equal to or surpassing 6 h. In contrast, only 5 studies adhered exclusively to the standards delineated by the Joint Commission International [76], which stipulates that a waiting time exceeding 4 h defines patient boarding. The dynamic nature of the ED presents challenges in standardizing processes [77]. This environment is indeed influenced by various factors, including crowding, the diversity of clinical cases treated, staff availability, and equipment provisioning [54]. Crowding in hospitals often surpasses the ED's capacity, resulting in longer wait times, treatment delays, and heightened pressure on healthcare staff [54]. The department manages a wide range of clinical cases, requiring adaptability and quick responses despite fluctuating staff availability and the crucial need for adequate equipment to diagnose and treat patients effectively [78, 79, 80]. Moreover, despite the included studies mostly referred to high‐income countries, which generally guarantee high‐standard in healthcare as reported by literature [81], the two included studies [67, 68] considering low‐ and middle‐income countries [82], did not demonstrate a statistically significant divergence in excess mortality. Moreover, Singer et al. (2011), the largest included study, reported an association between ≥ 2‐h boarding and increased mortality, which is not fully consistent with the overall findings of this synthesis. This heterogeneity likely reflects differences in study context, population structure, and methodological approaches, reinforcing the uncertainty surrounding the independent effect of boarding time on mortality. This further illustrates the multifactorial nature of the ED, which is inherently unpredictable based on currently available aggregated evidence [83]. Importantly, because different studies contributed to different duration categories, the between‐pool pattern cannot establish a mortality threshold or dose‐response relationship.

Within the literature, the prevailing view suggests the significance of prompt and proactive interventions by ED personnel as the fundamental strategy for averting complications linked to patient boarding [84, 85, 86, 87]. One potential support for the hypothesis that adequate patient management before accessing needed specialized care does not impact clinical deterioration is found in studies employing a 1‐h cut‐off [55, 57, 58, 65, 67], which show increased mortality rates in the non‐boarding group at the study level. In line with the extreme heterogeneity observed across the meta‐analysis (I^2^ = 99.9%), this pattern further supports that between‐pool differences are largely artefactual. This reflects underlying variability in populations, study designs, and case‐mix rather than true exposure effects, thereby limiting the validity of causal interpretation across subgroup estimates. Conversely, it is essential to assess the hypothesis that symptom severity may have facilitated preferential access to specialized treatment for patients who died without EDB [88]. Moreover, while there is broad clinical consensus supporting the importance of early intervention in emergency care, the evidence cited derives primarily from trauma and critical care settings, which are not fully aligned with the populations included in the present review [89]. Timely intervention is linked to improved outcomes in trauma [90], acute illnesses [91], and critical care scenarios [92], emphasizing the need for prompt assessment, diagnosis, and treatment to enhance survival rates and reduce complications [93], leading healthcare organizations to advocate prioritizing early intervention to optimize patient care and healthcare efficiency. It is possible that a general benefit of earlier management may extend, at least partially, to non–time‐sensitive conditions, even if this assumption remains indirect and not directly demonstrated in the present study.

Therefore, it is necessary to focus on training operators and implementing guidelines and best practices, as early stabilization of the patient would ensure a better outcome even in the face of longer wait times (as suggested by clinical literature, though not directly demonstrated in this analysis). The application of predictive algorithms eased using AI and/or IT support tools, whose adoption is currently economically favored by national regulations, could promote easier patient management. AI‐driven, data‐informed decision support systems already help trainees make knowledgeable and evidence‐based clinical choices in the emergency department [94]. Errors associated with inexperience, as from the employment of physicians during their specialization training, could be mitigated by implementing measures such as enhanced training protocols and supervision.

### 4.1 Limitations and Strengths

The lack of a universally accepted definition of EDB resulted in heterogeneity among studies, making a meta‐analysis unfeasible; instead, a pooled proportion approach was used. Variability in clinical frameworks and patient stratification by severity, along with the exclusion of time‐sensitive diseases [95] like myocardial infarction, may have introduced bias. Overall heterogeneity was extreme (I^2^ = 99.9%), indicating substantial between‐study variability and limiting the clinical interpretability of the pooled proportions.

Some studies contributed data to more than one boarding‐time subgroup when multiple thresholds were reported. Therefore, subgroup estimates should not be interpreted as fully independent comparisons. Additionally, publication bias could have overestimated the association between EDB duration and mortality, while the broader focus on all ED admissions raises concerns about generalizability. Moreover, different studies contributed to different thresholds, so differences between pooled proportions cannot be interpreted as threshold effects. A formal GRADE certainty‐of‐evidence assessment was not performed.

The study's strength lies in its rigorous methodology, including expert clinical review, quality appraisal, and data synthesis, ensuring internal validity. Excluding time‐sensitive scenarios reduced confounding factors, while standardized methods enhanced reliability.

### 4.2 Future Directions

Reliance on retrospective data limits causal inferences. Prospective studies are needed to better control confounders. Practical steps include targeted training, AI‐assisted screening protocols, and algorithm‐driven tools for ED‐bed management to support ED management and broader health system objectives, such as those related to national digital transformation and efficiency programs.

## 5 Conclusion

These results underscore the complexity of the relationship between boarding and mortality in the ED setting and emphasize the need for further research employing standardized methodologies and larger sample sizes to elucidate this relationship comprehensively. Understanding the relationship between boarding duration and mortality is crucial for informing ED management strategies and improving patient outcomes in this critical healthcare setting. The threshold‐specific pooled proportions are descriptive, do not demonstrate a causal or dose‐response relationship, and should be interpreted cautiously given the extreme heterogeneity (I^2^ = 99.9%).

## Author Contributions

**Gerardo Altamura:** conceptualization, investigation, writing – original draft, writing – review and editing, data curation. **Mario Cesare Nurchis:** investigation, validation, formal analysis, data curation, methodology, resources. **Silvia Martinelli:** writing – original draft, writing – review and editing, data curation, resources. **Annarita Panebianco:** investigation, methodology, writing – review and editing, supervision. **Gabriele Giubbini:** writing – original draft, data curation. **Marcello Covino:** conceptualization, validation, data curation. **Francesco Franceschi:** conceptualization, supervision, visualization, validation. **Gianfranco Damiani:** conceptualization, validation, data curation, resources.

## Disclosure

All authors have read and approved the final version of the manuscript. The corresponding author (Dr. Gerardo Altamura) had full access to all data in this study and takes full responsibility for the integrity of the data and the accuracy of the data analysis.

## Funding

The authors have nothing to report.

## Ethics Statement

The authors have nothing to report.

## Conflicts of Interest

The authors declare no conflicts of interest.

## Transparency Statement

The corresponding author (Gerardo Altamura) confirms that this proportional meta‐analysis is a truthful, accurate, and transparent representation of the work undertaken. All relevant methodological and analytical elements have been fully reported, and no important aspects have been omitted. As this study is a proportional meta‐analysis, preregistration and protocol deviation assessments are not applicable.

## Supporting information


Supporting File 1

Supporting File 2

Supporting File 3

Supporting File 4

## Data Availability

All data generated or analysed during this study are available from the authors upon reasonable request. Researchers interested in accessing the dataset may contact the corresponding author for further information.
