# Supplementary material for: Emergency Department Boarding and Mortality: A Systematic Review and Proportional Meta‐Analysis
Source: Health Sci Rep. 2026 Nov 4;9(11):e73400. doi: 10.1002/hsr2.73400 (PMC13732418; doi:10.1002/hsr2.73400)

Study	Events	Total	Proportion	95%-CI	Weight
**2_No boarding**					
Singer, 2011	513	20527	0.025	[0.023; 0.027]	3.0%
**6_Boarding**					
Singer, 2011	409	15145	0.027	[0.024; 0.030]	3.0%
Lin, 2022	690	5056	0.136	[0.127; 0.146]	3.0%
Gardner, 2022	82	612	0.134	[0.108; 0.164]	3.0%
Chalfin, 2007	180	1036	0.174	[0.151; 0.198]	3.0%
Huang, 2024	118	477	0.247	[0.210; 0.289]	2.9%
Intas, 2012	88	140	0.629	[0.543; 0.708]	2.8%
Nesbitt, 2021	51	506	0.101	[0.075; 0.130]	3.0%
Puls, 2022	593	4767	0.124	[0.115; 0.134]	3.0%
Bing-Hua, 2014	16	185	0.086	[0.053; 0.139]	2.8%
**Random effects model**		**27924**	**0.162**	**[0.097; 0.240]**	**26.5%**
Heterogeneity: $I^2 = 99.5\%$, $p = 0$					
**12_Boarding**					
Singer, 2011	122	3121	0.039	[0.032; 0.046]	3.0%
Verma, 2021	138	282	0.489	[0.430; 0.549]	2.9%
**Random effects model**		**3403**	**0.218**	**[0.000; 0.753]**	**5.9%**
Heterogeneity: $I^2 = 99.7\%$, $p < 0.001$					
**24_Boarding**					
Singer, 2011	94	2086	0.045	[0.037; 0.055]	3.0%
Groenland, 2019	329	2940	0.112	[0.101; 0.124]	3.0%
Verma, 2021	274	532	0.515	[0.472; 0.558]	3.0%
**Random effects model**		**5558**	**0.190**	**[0.042; 0.409]**	**9.0%**
Heterogeneity: $I^2 = 99.7\%$, $p < 0.001$					
**6_No boarding**					
Lin, 2021	1144	8578	0.133	[0.126; 0.141]	3.0%
Gardner, 2022	236	1250	0.189	[0.168; 0.212]	3.0%
Chalfin, 2007	6358	49286	0.129	[0.126; 0.132]	3.0%
Huang, 2024	162	841	0.193	[0.165; 0.219]	3.0%
Intas, 2012	28	60	0.467	[0.336; 0.600]	2.5%
Nesbitt, 2021	229	1730	0.132	[0.117; 0.149]	3.0%
Puls, 2022	12	1090	0.011	[0.005; 0.019]	3.0%
Bing-Hua, 2014	140	2094	0.067	[0.050; 0.072]	3.0%
**Random effects model**		**64929**	**0.130**	**[0.099; 0.165]**	**23.5%**
Heterogeneity: $I^2 = 98.5\%$, $p < 0.001$					
**1_No boarding**					
Tantarattanapong, 2021	39	358	0.109	[0.077; 0.144]	2.9%
Peltan, 2019	565	6158	0.092	[0.084; 0.099]	3.0%
Groenland, 2019	616	2956	0.208	[0.193; 0.223]	3.0%
**Random effects model**		**9472**	**0.133**	**[0.060; 0.229]**	**9.0%**
Heterogeneity: $I^2 = 99.1\%$, $p < 0.001$					
**1_Boarding**					
Tantarattanapong, 2021	22	242	0.091	[0.059; 0.136]	2.9%
Peltan, 2019	322	4653	0.069	[0.062; 0.077]	3.0%
**Random effects model**		**4895**	**0.074**	**[0.056; 0.093]**	**5.9%**
Heterogeneity: $I^2 = 40.8\%$, $p = 0.194$					
**2_Boarding**					
Groenland, 2019	619	2998	0.206	[0.192; 0.221]	3.0%
**4_Boarding**					
Groenland, 2019	1119	5894	0.190	[0.178; 0.199]	3.0%
**8_No boarding**					
Hong, 2009	13	140	0.093	[0.053; 0.156]	2.8%
**8_Boarding**					
Hong, 2009	15	55	0.273	[0.167; 0.411]	2.4%
Verma, 2021	369	683	0.540	[0.502; 0.578]	3.0%
**Random effects model**		**738**	**0.412**	**[0.172; 0.676]**	**5.4%**
Heterogeneity: $I^2 = 93.4\%$, $p < 0.001$					
**4_No boarding**					
Verma, 2021	848	1653	0.513	[0.489; 0.537]	3.0%
**Random effects model**		**148131**	**0.168**	**[0.135; 0.204]**	**100.0%**

Heterogeneity: $I^2 = 99.6\%$, $p = 0$

Test for subgroup differences: $\chi^2_{11} = 4148.46$, df = 11 ($p = 0$)

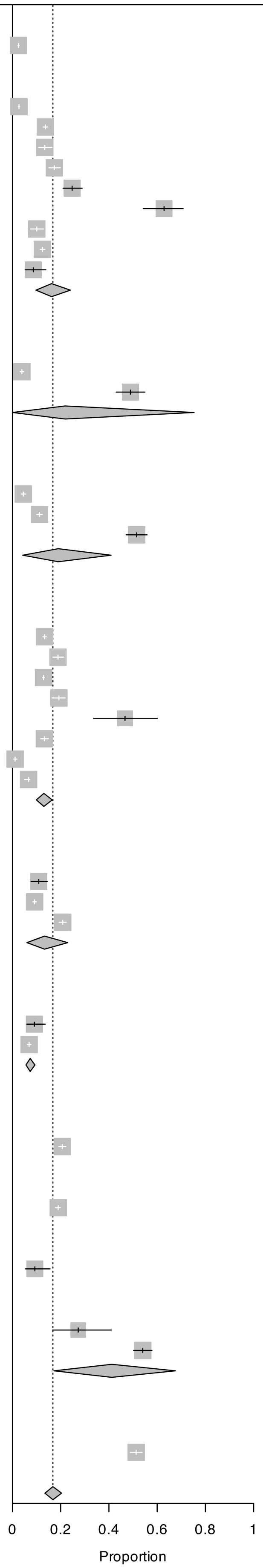

Supplement: Supplementary file 1 [file HSR2-9-e73400-s001.pdf]
