# Supplementary material for: Emergency Department Boarding and Mortality: A Systematic Review and Proportional Meta‐Analysis
Source: Health Sci Rep. 2026 Nov 4;9(11):e73400. doi: 10.1002/hsr2.73400 (PMC13732418; doi:10.1002/hsr2.73400)
Supplement: Supplementary file 2 [file HSR2-9-e73400-s002.pdf]

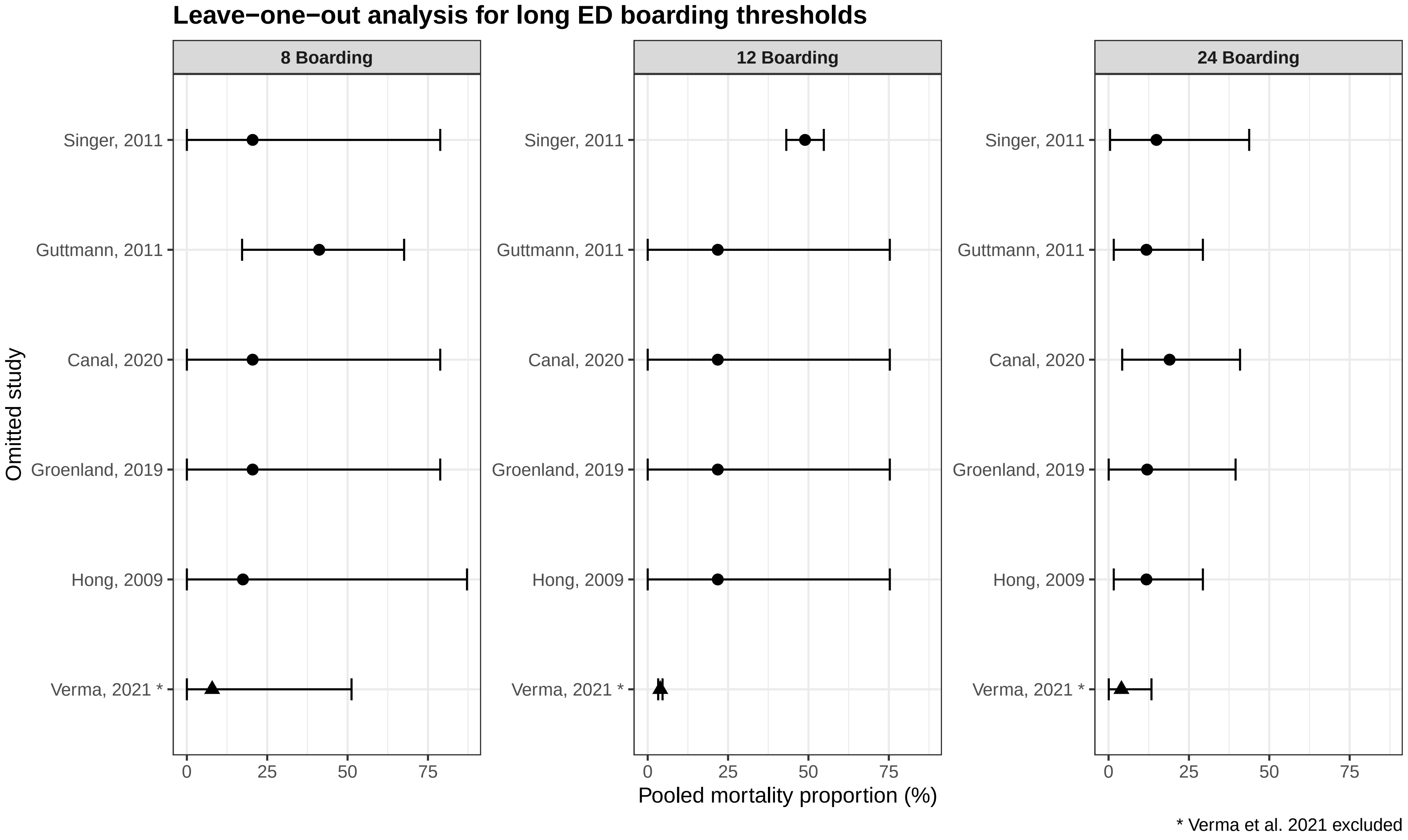
Leave−one−out analysis for long ED boarding thresholds
8 Boarding
12 Boarding
24 Boarding
Omitted study
Singer, 2011
Guttmann, 2011
Canal, 2020
Groenland, 2019
Hong, 2009
Verma, 2021 *
0
25
50
75
Pooled mortality proportion (%)
* Verma et al. 2021 excluded
