# Supplementary material for: Emergency Department Boarding and Mortality: A Systematic Review and Proportional Meta‐Analysis
Source: Health Sci Rep. 2026 Nov 4;9(11):e73400. doi: 10.1002/hsr2.73400 (PMC13732418; doi:10.1002/hsr2.73400)
Supplement: Supplementary file 3 [file HSR2-9-e73400-s003.pdf]

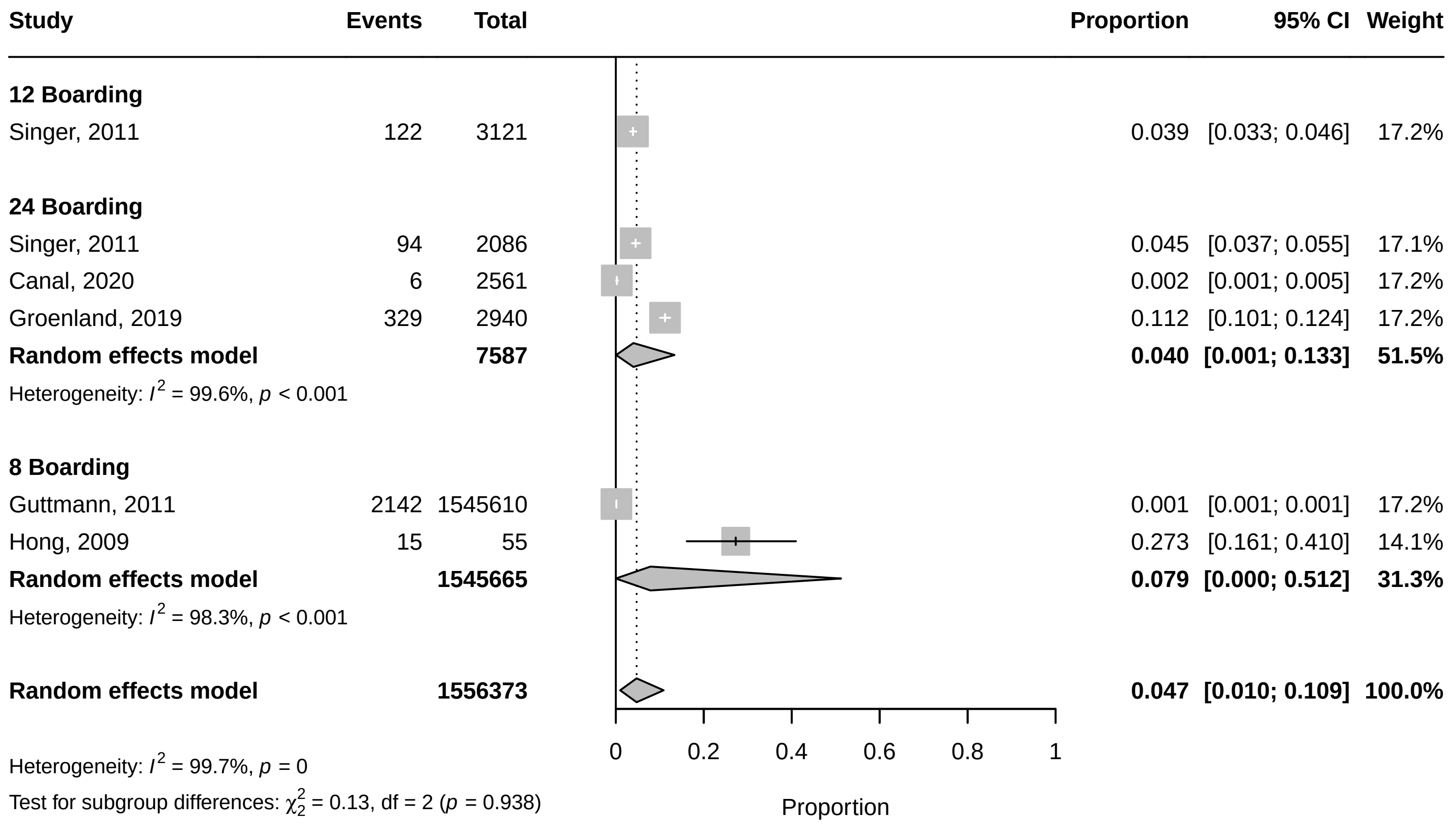

Study Events Total Proportion 95% CI Weight
12 Boarding
Singer, 2011 122 3121 0.039 [0.033; 0.046] 17.2%
24 Boarding
Singer, 2011 94 2086 0.045 [0.037; 0.055] 17.1%
Canal, 2020 6 2561 0.002 [0.001; 0.005] 17.2%
Groenland, 2019 329 2940 0.112 [0.101; 0.124] 17.2%
Random effects model 7587 0.040 [0.001; 0.133] 51.5%
Heterogeneity: I^2 = 99.6%, p < 0.001
8 Boarding
Guttmann, 2011 2142 1545610 0.001 [0.001; 0.001] 17.2%
Hong, 2009 15 55 0.273 [0.161; 0.410] 14.1%
Random effects model 1545665 0.079 [0.000; 0.512] 31.3%
Heterogeneity: I^2 = 98.3%, p < 0.001
Random effects model 1556373 0.047 [0.010; 0.109] 100.0%
Heterogeneity: I^2 = 99.7%, p = 0
Test for subgroup differences: χ^2_2 = 0.13, df = 2 (p = 0.938)
0 0.2 0.4 0.6 0.8 1
Proportion
